# Quantification of nitroaromatic explosives in contaminated soil using MALDI-TOF mass spectrometry

**DOI:** 10.1007/s00216-019-01976-y

**Published:** 2019-07-05

**Authors:** S. Liane Kober, Henner Hollert, Marcus Frohme

**Affiliations:** 1 Molecular Biotechnology and Functional Genomics, Technical University of Applied Sciences Wildau, Hochschulring 1, 15745 Wildau, Germany; 2 Institute for Environmental Research (Biology V), Department of Ecosystem Analysis, RWTH Aachen University, Worringerweg 1, 52074 Aachen, Germany

**Keywords:** Explosives, Soil contamination, TNT, Matrix-assisted laser desorption/ionization quantification

## Abstract

Contamination from various sources is a global environmental and health threat, with mining and military activities in particular having spread nitroaromatic compounds, such as 2,4,6-trinitrotoluene and its degradation products and by-products, to the soil. The investigation and monitoring of large contaminated areas requires new detection methods since the established ones are expensive and time-consuming. Hence, we established a matrix-assisted laser desorption/ionization time-of-flight mass spectrometry (MALDI–TOF MS) method using 1,5-diaminonaphthalene as the matrix substance and an internal standard for quantification. Analyzing standard substances, we found specific signals for radical and fragment ions of different nitrotoluenes and nitrobenzenes with good reproducibility and detection limits down to 0.25 ng/μL. The analysis of soil sample extracts from a former production site showed clear signals for 2,4,6-trinitrotoluene and the primary degradation products aminodinitrotoluenes. Furthermore, quantification gave results comparable to those obtained by conventional liquid chromatography–tandem mass spectrometry analysis. The MALDI-TOF MS method has a comparatively lower reproducibility, with relative standard deviations of 6% to 20% for multiple measurements of standard solutions and soil sample extracts. Nevertheless, a comparison of both methods revealed the advantages of MALDI-TOF MS analysis of explosive-contaminated areas with regard to costs, time, and handling. Finally, our MALDI-TOF MS method fulfills all the needs for high sample throughput and can therefore be a valuable screening tool for explosive-contaminated areas.

**Electronic supplementary material:**

The online version of this article (10.1007/s00216-019-01976-y) contains supplementary material, which is available to authorized users.

**Figure:**
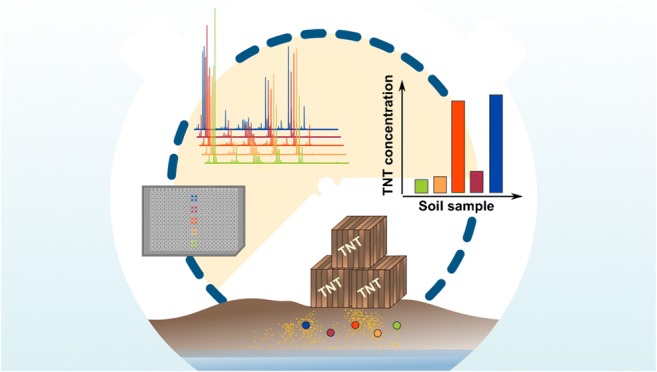
Graphical abstract

## Introduction

The discovery of gunpowder opened new perspectives for industrial and military applications. Further improvements were achieved through the targeted synthesis of explosives, which increased not only the destruction potential but also their use as well as their subsequent environmental distribution as contaminants. Specifically, 2,4,6-trinitrotoluene (TNT) rapidly dominated the explosives industry and became one of the most commonly used explosives in World War I and World War II [1]. The large-scale production of up to 2.34 × 10^4^ tons TNT per month and its use, testing, and finally improper disposal resulted in vast areas that are still contaminated with TNT [2]. The US Army estimated a total of 1.2 million tons of contaminated soil in the USA [2, 3]. Similar magnitudes of contamination can be found in other countries, being the result of unexploded ordnance on the scale of hundreds of thousands, millions of landmines, and numerous production and testing sites [1, 3]. Soil concentrations of up to 87,000 ppm TNT [1, 4] and even 600,000–700,000 ppm TNT [5] are stated in the literature, and not only do these threaten the health of humans but they are also a major concern for entire ecosystems [6]. TNT is toxic, mutagenic, a potential carcinogen, and environmentally persistent [3, 7–10]. The explosive contamination spreads from the contamination source, reaches groundwater levels, binds to soil organic matter, and is transformed and degraded, depending highly on soil characteristics and soil microbiota [3].

Ion mobility spectrometry is often applied for the field detection of explosives and is widely used for the detection of trace levels of nitroaromatic explosives at airports [11]. Small handheld and user-friendly devices are available that allow real-time monitoring and have higher sensitivity than mass spectrometry (MS) technologies [12]. On the other hand, ion mobility spectrometry has lower selectivity, a limited linear range, and low resolution [12]. Besides, high concentrations of explosives and complex matrices may lead to saturation and contamination of the instrument, which causes interferences in quantitative determinations [12, 13]. Most commonly, high-performance liquid chromatography (HPLC) with UV detection or MS is used for the quantification of nitroaromatic explosives on contaminated sites [14]. Despite the advantage of very sensitive detection, liquid chromatography (LC)–MS is associated with high costs and lengthy sample analysis and requires well-qualified staff [14]. Conversely, matrix-assisted laser desorption/ionization (MALDI) time-of-flight (TOF) MS is simple, tolerant of sample impurities, and fast, needs small sample volumes, and can be automated easily, which is a fundamental requirement for high-throughput applications [15]. Originally, MALDI-TOF MS was most frequently used for the qualitative analysis of high molecular weight compounds, such as proteins, peptides, polymers, and even prokaryotic or eukaryotic cells [15, 16]. In contrast, the analysis of low molecular weight compounds or quantification of substances was not the focus of research because the organic matrix often leads to the suppression of analyte peaks in the low mass range [15]. However, in recent years several approaches using new matrix substances increased the applicability of MALDI-TOF MS for small molecules, such as oligosaccharides, phospholipids, peptides, metabolites, drugs, and environmental contaminants [17–22]. Furthermore, several studies identified the key factors to allow the MALDI-TOF MS quantification of analytes, namely, thorough premixing of sample and matrix solutions, fast crystallization times (e.g., by the use of prestructured target surfaces), the acquisition of multiple spectra from different spots on the target, and the use of a structurally similar internal standard [23, 24]. Because of the unique properties of MALDI-TOF MS and the need for large-scale investigations of areas contaminated with explosives, we developed a method for the detection and quantification of nitroaromatic compounds using an internal standard. Among conventional matrices, we used 1,5-diaminonaphthalene (DAN), which showed excellent properties in previous studies for the analysis of phospholipids and several metabolites [21, 25]. DAN should be handled with care as it is assumed to be carcinogenic [21]. We applied DAN successfully for the analysis of nitroaromatic explosives as single compounds and in mixtures. With the preparation of calibration curves, we were able to quantify the contamination of soil samples from a former TNT production site using MALDI-TOF MS and compared the results with those obtained by conventional LC–MS/MS analysis.

## Materials and methods

### Reagents/chemicals

Solvents for LC–MS/MS, such as acetonitrile (ACN), methanol, and water were HPLC or MS grade and were obtained from Carl Roth (Karlsruhe, Germany). All other chemicals used were HPLC or analytical grade (p.a.). The solvent additives ammonium hydroxide and ammonium acetate were purchased from Carl Roth and Honeywell Riedel-de Haën (Seelze, Germany), respectively. Matrices for MALDI-TOF MS, such as DAN, 9-aminoacridine (AA), α-cyano-4-hydroxycinnamic acid (CHCA), 2,4,6-trihydroxyacetophenone (THAP), and multiwalled carbon nanotubes, as well as standard explosive solutions of 2-amino-4,6-dinitrotoluene (2-ADNT), 4-amino-2,6-dinitrotoluene (4-ADNT), 1,3,5-trinitrobenzene (TNB), 1,3-dinitrobenzene (DNB), 2-nitrotoluene (NT), and 5-chloro-2,4-dinitrotoluene (CDNT) were obtained from Sigma-Aldrich (St Louis, MO, USA). TNT, 2,4-dinitrotoluene (2,4-DNT), and 2,6-dinitrotoluene (2,6-DNT) were provided by terracon (Jüterbog, Germany). Mefenamic acid and chlorogenic acid were from Sigma-Aldrich. Citric acid was purchased from Merck (Darmstadt, Germany). Standard stock solutions were prepared as a mixture of each explosive at 50 ng/μL (TNT, 2-ADNT, 4-ADNT, 2,4-DNT, 2,6-DNT, TNB, DNB, NT) in ACN for LC–MS/MS measurements. For MALDI-TOF MS, a stock solution consisting of TNT, TNB, DNB, NT, 1:10 2-ADNT/4-ADNT, and 1:1 2,4-DNT/2,6-DNT each at 100 ng/μL in ACN was used.

### Soil sample preparation

Soil samples originated from a former TNT production site in Brandenburg (Germany) and were provided by terracon. A total of 5–10 kg of each soil sample was collected and mixed thoroughly, and subsamples were used for MALDI-TOF MS and LC–MS/MS analysis. The extraction was based on Environmental Protection Agency method 8330b [26] and the well-established method of our industrial partner, respectively. It was done according to our previously published and validated method [27]. Briefly, approximately 2 g of each soil sample was mixed with 4 mL ACN and extracted for at least 18 h on an overhead shaker (Intelli-Mixer RM-2, ELMI, Riga, Latvia). Extracts were centrifuged at 5000*g* for 2 min, and supernatants were filtered through 0.22-μm polytetrafluoroethylene filters with use of disposable syringes. Before LC–MS/MS analysis, extracts were diluted with ACN.

### LC–MS/MS

The LC–MS/MS system (LCMS-8040, Shimadzu, Manchester, UK) consisted of an HPLC system and a triple-quadrupole mass spectrometer. We successively optimized solvents, the gradient, the flow rate, and the column temperature to achieve a good peak separation and fast run time [27]. HPLC separation was performed with a Nucleodur C_18_ HTec column (150 mm × 2 mm, 3 μm; Macherey-Nagel, Düren, Germany) at a flow rate of 0.15 mL/min and a column temperature of 28 °C in gradient mode with water (mobile phase A) and methanol (mobile phase B). The starting methanol concentration was 57% (0–2 min). The methanol concentration was increased to 70% (2–13 min), maintained at 70% (13–14.8 min), reduced to 10% (14.8–15 min), and finally kept at 10% (15–20 min). UV detection and quantification were done at 254 nm. The mass spectrometer was mass-calibrated against a standard sample for LC–MS (Shimadzu). To increase the efficiency of the electrospray ionization (ESI), 5 mM ammonium acetate and 0.001% ammonium hydroxide were added to mobile phase A. For mass detection, negative-ion ESI MS/MS was used to characterize product and specific fragment ions of the explosives (multiple reaction monitoring). The technical parameters for the MS measurements were a spray capillary voltage of 3.0 kV, a detector voltage of 2.04 kV, a desolvation line temperature of 250 °C, a heat block temperature of 450 °C, a nebulizing gas flow rate of 3.0 mL/min, a drying gas flow rate of 15 mL/min, and a collision-induced dissociation gas pressure of 230 kPa. Data analysis was performed with LabSolutions (version 5.65, Shimadzu). Retention times, precursor and product ions, and individual collision energies for the five detectable explosives can be found in Table 1. Calibration curves were prepared by serial dilution of the mixture of explosives and were measured in the range from 0.001 to 10 ng/μL before sample analysis. The injection volume of the standards and samples was 1 μL.

**Table 1:** Liquid chromatography–tandem mass spectrometry parameters for the detection of the explosives 1,3,5-trinitrobenzene (TNB), 2,4,6-trinitrotoluene (TNT), 4-amino–2,6-dinitrotoluene (4-ADNT), 2-amino–4,6-dinitrotoluene (2-ADNT), and 2,4-dinitrotoluene (2,4-DNT)

Analyte	Retention time (min)	Precursor ion *m*/*z*	Product ion *m*/*z*	Collision energy (V)
TNB	8.3	183.10	95.10	20
TNT	10.9	226.10	196.10	11
4-ADNT	11.2	196.10	149.15	14
2-ADNT	11.6	196.10	150.10	18
2,4-DNT	12.2	181.10	116.05	14

### MALDI-TOF MS

Different approaches and matrices (CHCA, THAP, multiwalled carbon nanotubes, AA, DAN) were successfully applied for the detection of TNT and ADNT. Matrix solvents, concentrations (10 μg/mL to 20 mg/mL), and application (co-crystallization, layer-by-layer) were optimized individually. The final approach used DAN and the internal standard CDNT and was conducted as follows: Standard mixtures of explosives and soil sample extracts were mixed 1:1 with the matrix (DAN, 20 mg/mL, in 80:20 ACN/water) including the internal standard (CDNT at 25 ng/μL). Then 1 μL of the mixture was deposited onto the MALDI-TOF MS target in triplicate and air-dried to form homogeneous spot crystals. Mass calibration was performed with a self-made calibration mixture of matrix, mefenamic acid (100 ng/μL), citric acid (100 ng/μL), chlorogenic acid (100 ng/μL), and CDNT (100 ng/μL).

MALDI-TOF mass spectra were obtained with an Axima Confidence mass spectrometer (Shimadzu Biotech, Manchester, UK) equipped with a 337-nm nitrogen laser. The filter for regulation of the laser firing power was set to 20. To generate representative profiles, 250 laser shots were accumulated and averaged for each spot. Mass spectra were recorded in negative high-resolution reflectron mode at a maximal laser repetition rate of 50 Hz within a mass range of 120 to 300 Da.

Data processing was performed with Launchpad (version 2.9, Shimadzu Biotech) as well as with the statistical computing language R with the packages MALDIquant, ggplot2, and factoextra [28–31]. All MALDI-TOF mass spectra shown were processed (i.e., aligned and normalized) according to the total ion current. Two-way ANOVA was performed with Microsoft Excel 2016.

## Results and discussion

### MALDI-TOF MS of explosives

To achieve good ionization, the choice of the matrix is crucial. MALDI-TOF MS methods using CHCA and sinapic acid and other laser TOF MS methods have been published for the detection of different explosives (TNT, RDX, HMX, DNT, trinitrophenol) [32–35]. However, the detection of ADNT, the direct quantification of explosives, or the analysis of complex environmental soil extracts was not attempted.

**Fig. 1:**
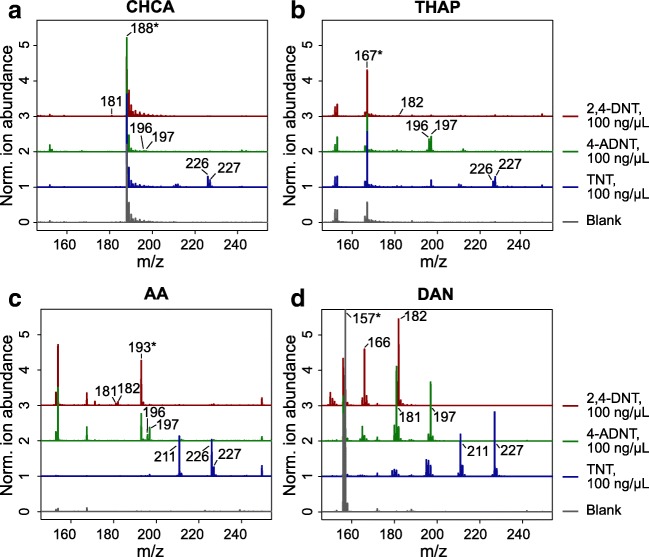
Different matrix-assisted laser desorption/ionization **(**MALDI) matrices that were tested and optimized for the detection of the nitroaromatic explosives 2,4–dinitrotoluene (2,4-DNT), 4-amino-2,6-dinitrotoluene (4-ADNT) and 2,4,6-trinitrotoluene (TNT): **a** α-cyano-4-hydroxycinnamic acid (CHCA; 100 μg/mL) in 70:30 acetonitrile (ACN)/H_2_O plus 0.1% trifluoroacetic acid (TFA); **b** 2,4,6-trihydroxyacetophenone (THAP; 100 μg/mL) in 70:30 ACN/H_2_O plus 0.1% TFA; **c** 9-aminoacridine (AA; 100 μg/mL) in 70:30 ACN/H_2_O; **d** 1,5-diaminonaphthalene (DAN; 20 mg/mL) in 80:20 ACN/H_2_O. Characteristic product and fragment ions as well as matrix ions (asterisk) are labeled. Each spectrum is the average of three replicates. The offset between spectra is 1.

We tested different matrices, optimized their composition, and found four matrices (CHCA, THAP, AA, and DAN) that were suited for the analysis of three selected explosive standards (TNT, 4-ADNT, and 2,4-DNT). For most matrices, either deprotonated [M − H]^-^ or radical [M]^•-^ anions were produced (Fig. 1). CHCA, THAP, or AA demonstrated only weak signals for ions of 2,4-DNT (*m*/*z* 181, *m/z* 182), but supported the ionization of TNT (*m*/*z* 226, *m*/*z* 227) and partly that of 4-ADNT (*m*/*z* 196, *m*/*z* 197). To enhance analyte signals we used the matrix-suppression effect (i.e., the suppression of matrix molecules through low laser intensities and a favorable analyte/matrix ratio) [32]. The greatest matrix-suppression effect and highest sensitivity were obtained with matrix concentrations of 100 μg/mL and co-crystallization of analyte/sample mixtures for all matrices tested.

Other prominent approaches have included the use of a high molecular weight matrix [36], additives for the reduction of matrix-related background [37], or ionic liquid matrices [38], which were not successful for the analysis of nitroaromatic explosives. Nevertheless, the small difference in molecular mass between ADNT (197 g/mol) and the matrix substances CHCA (188 g/mol) and AA (194 g/mol), respectively, probably resulted in a suppression effect. Furthermore, none of the three matrices were suitable for DNT analysis.

However, another way to analyze low molecular weight compounds is the eschewal of a matrix and the use of a photoactive support such as carbon nanotubes [39, 40]. We were able to analyze and establish calibration curves using multiwalled carbon nanotubes (data not shown), but this led to contamination of the ion source of the mass spectrometer. We do not recommend using carbon nanotubes for MALDI-TOF MS without proper immobilization [40]. By far the best results were obtained with DAN as the matrix (Fig. 1d). Not only radical anions but also specific fragments were found (e.g., corresponding to *m*/*z* 227 and *m*/*z* 211 for TNT). Low DAN concentrations were also suited for MALDI-TOF MS of standard explosive solutions. Environmental samples (soil extracts) needed a much higher laser intensity, probably because of soil matrix effects. Hence, we decided to use a high matrix concentration of 20 mg/mL, which resulted in a slight decrease of sensitivity but allowed uniform measurement settings with a low laser intensity and provided good results for further investigations. The use of highly concentrated explosive standards (100 ng/μL) produced strong and clear signals, making it likely we would detect even low levels of contamination in soil. Precursor ions of DAN with *m*/*z* 156 and *m*/*z* 157 were detected. It was speculated previously that these masses occur due to reduction reactions involving the amino groups of DAN [21]. Reductive and in-source decay (ISD) properties of the matrix [41, 42] may also lead to the partial conversion of the explosives’ nitro groups to nitroso groups, which resulted in the observed fragments with a mass shift of *m/z* 16. This reductive transformation of nitroaromatic explosives is a well-known natural phenomenon. Because of the high electron deficiency of the ring *π* system, nitroaromatic compounds are primarily transformed through reduction [43].

**Fig. 2:**
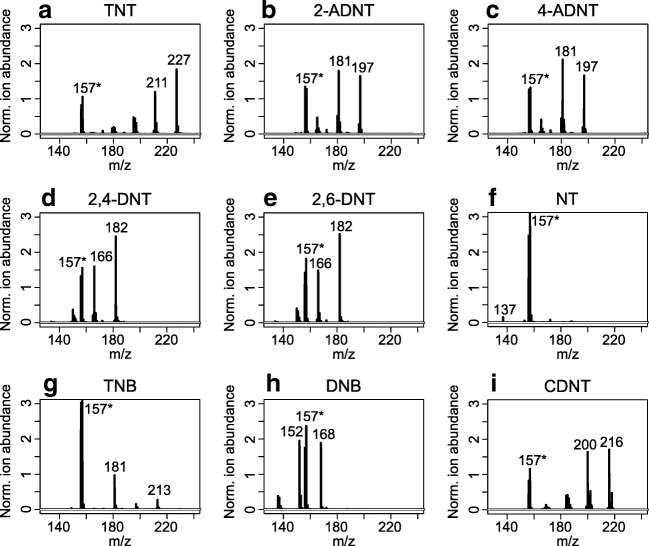
Matrix-assisted laser desorption/ionization time-of-flight mass spectrometry analysis of different nitroaromatic compounds each at a concentration of 100 ng/μL: **a** 2,4,6-trinitrotoluene (TNT); **b** 2-amino-4,6-dinitrotoluene (2-ADNT); **c** 4-amino-2,6-dinitrotoluene (4-ADNT); **d** 2,4-dinitrotoluene (2,4-DNT); **e** 2,6-dinitrotoluene (2,6-DNT); **f** 2-nitrotoluene (NT); **g** 1,3,5-trinitrobenzene (TNB); **h** 1,3-dinitrobenzene (DNB); **i** 5-chloro-2,4-dinitrotoluene (CDNT). Characteristic product and fragment ions as well as matrix ions (asterisk) are labeled (1,5-diaminonaphthalene, 20 mg/mL, in 80:20 acetonitrile/water, negative high-resolution reflectron mode). Each spectrum is the average of three replicates.

Following this, the ionization properties of the DAN matrix for further nitroaromatic compounds, namely, 2-ADNT, 2,6-DNT, NT, TNB, and DNB, were investigated. Furthermore, MALDI-TOF MS of CDNT was studied for the applicability of CDNT as an internal standard. Most of the explosive standards exhibited good ionization properties and signals for radical anions as well as fragment ions (Fig. 2). Only NT exhibited poor ionization and a low signal of a radical anion (Fig. 2f).

The analysis of the isomers of ADNT and DNT led to similar mass spectra: 2-ADNT and 4-ADNT (Fig. 2b,c) both produced signals corresponding to *m*/*z* 197 and *m*/*z* 181, and 2,4-DNT and 2,6-DNT (Fig. 2d,e) showed ions with *m*/*z* 182 and *m*/*z* 166. Thus, the distinction of isomers with the MALDI-TOF MS method presented is not possible and the analysis of environmental samples will always result in the isomer sums of aminodinitrotoluenes, dinitrotoluenes, or nitrotoluenes, respectively. MALDI MS/MS may be suited for this purpose, but opportunities and limits for the analysis of explosives still have to be investigated [44].

We were able to detect 2,6-DNT, DNB, and NT (Fig. 2e,f,h), whereas this was not possible with LC–ESI MS/MS. The physical properties of the ionization source influence the selectivity for the analyzed compounds [45]. Whereas ion–molecule reactions in ESI occur in the liquid phase [45], theories on MALDI ion generation are based on gas-phase chemistry [46]. MALDI-TOF MS of CDNT produced clear signals of radical anions (*m*/*z* 216) and fragment ions (*m*/*z* 200), which do not interfere with other explosive-related signals. Besides, CDNT is not used for explosives but has structural similarity and is not expected to be found in the environment. Often stable-isotope-labeled internal standards are used instead of chemical analogues [24], but they are more expensive. Hence, CDNT was chosen as an internal standard for further investigations.

### Concentration dependency of explosive signals

**Fig. 3:**
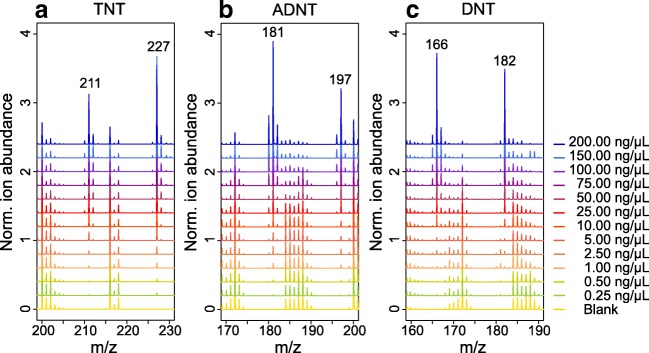
Matrix-assisted laser desorption/ionization time-of-flight mass spectrometry analysis of 2,4,6-trinitrotoluene (TNT) and 1:10 2-amino-4,6-dinitrotoluene/4-amino-2,6-dinitrotoluene (ADNT) and 1:1 2,4-dinitrotoluene/2,6-dinitrotoluene (DNT) mixtures in different explosive concentrations with the matrix (1,5-diaminonaphthalene, 20 mg/mL, in 80:20 acetonitrile/water) and the internal standard 5-chloro-2,4-dinitrotoluene (25 ng/μL) in negative high-resolution reflectron mode. The spectra reveal concentration-dependent signals of parent and fragment ions. Each spectrum is the average of three replicates. The offset between spectra is 0.2

The signals of the compounds of interest can be found in a small mass range between 130 and 230 Da. As a consequence, the interdependency of the different explosives’ ions and fragments in a complex sample can influence the correct identification and even more importantly the quantification. For that reason, we tested our method with different concentrations of the nitroaromatic explosives TNT, ADNT, and DNT, which can be found most frequently on contaminated sites. For ADNT and DNT we used mixtures of 1:10 2-ADNT/4-ADNT and 1:1 2,4-DNT/2,6-DNT. From our experience those proportions can be often found in contaminated areas. The MALDI-TOF MS spectra of all three explosives showed concentration-dependent signals for the specific explosive ions (Fig. 3). Even a very low concentration of 0.25 ng/μL produced signals for TNT or ADNT (Fig. 3a,b). To create a distinct signal of DNT, at least 1 ng/μL is needed (Fig. 3c). All deposited solutions contained a constant concentration of the internal standard CDNT. The corresponding signals maintained nearly uniform ion abundances in all spectra, which can be seen as the signals at *m*/*z* 216 and *m*/*z* 200 in panel a in Fig. 3 and is a good precondition for quantification purposes.

To prove that the determined peaks are specific for the single explosives, we conducted principal component analysis of all peaks of interest and their ion abundance based on the mass spectra shown in Fig. 3. Increasing concentrations of the individual explosives resulted in distinct mass spectra, which can be found in separate corners of the plot, thus indicating explosive-specific signals (see Fig. S2).

### Calibration and quantification using simple linear regression

**Fig. 4:**
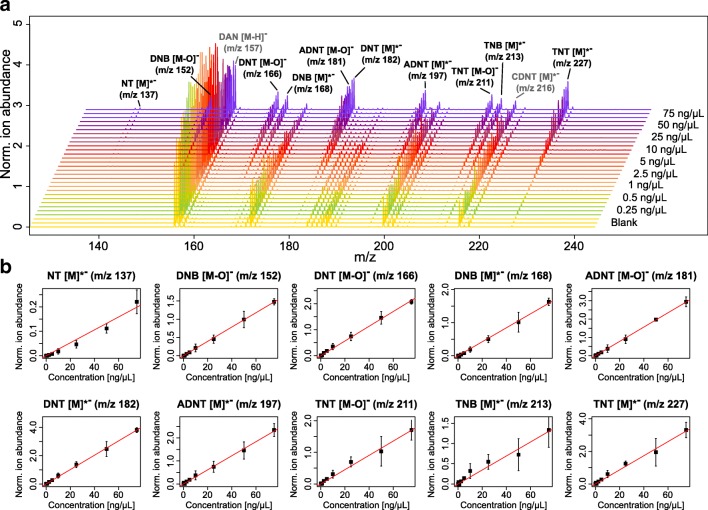
**a** Matrix-assisted laser desorption/ionization time-of-flight mass spectrometry analysis of mixtures of explosives in concentrations ranging from 0 (blank) to 75 ng/μL containing 2-nitrotoluene (NT), 1,3-dinitrobenzene (DNB), 1:1 2,4-dinitrotoluene/2,6-dinitrotoluene (DNT), 1:10 2-amino-4,6-dinitrotoluene/4-amino-2,6-dinitrotoluene (ADNT), 1,3,5-trinitrobenzene (TNB), and 2,4,6-trinitrotoluene (TNT) using the matrix 1,5-diaminonaphthalene (DAN; 20 mg/mL in 80:20 acetonitrile/water) and the internal standard 5-chloro-2,4-dinitrotoluene (CDNT; 25 ng/μL) in negative high-resolution reflectron mode (***n*** = 3). **b** Corresponding calibration curves for distinct peaks after normalization to the internal standard (CDNT [M]^**•**-^, *m***/***z* 216)

If soil is contaminated with nitroaromatic explosives, the extracts often contain not only one compound but rather several degradation products. Their parallel identification and quantification with conventional chromatographic methods is often achieved with a mixture of several standard substances, which is used for calibration. Subsequently, explosives in soil extracts are quantified on the basis of their specific retention time and mass if a mass spectrometer is at hand. We followed a similar approach and used MALDI-TOF MS to generate spectra of a mixture of standard explosives in different concentrations. In the resulting spectra all explosive components were identified, revealed concentration-dependent signals, and showed good reproducibility for the measurement of replicates (Fig. 4a). On the basis of this, the explosive-specific signals were normalized to the ion abundance of the internal standard (*m*/*z* 216) and plotted against the concentration of the explosive (Fig. 4b). We then performed classical linear least squares regression. The resulting calibration curves exhibit good linearity in the calibration range. Only the plots for *m*/*z* 137 ([NT]^•-^]), *m*/*z* 211 ([TNT − O]^•-^), *m*/*z* 213 ([TNB]^-^), and *m*/*z* 227 ([TNT]^•-^) partly have a higher standard deviation. As shown before, the detection of NT is not very sensitive. The masses of [TNT − O]^•-^ and [TNB]^-^ are quite similar, which may be a reason for increased ion suppression or interferences through naturally occurring isotopes [19]. However, the simple linear regression accurately reflects the concentrations used for all *m*/*z* investigated. Hence, it was used to calculate the explosives’ concentrations in contaminated soil extracts. Multiple measurements of a standard solution and two soil extracts showed good reproducibility for the quantification of ADNT and TNT (Fig. S3). The relative standard deviation ranged from 6% to 20%. Conversely, weak contamination of the soil extracts with DNT exhibited a high relative standard deviation of up to 50%. However, there was significant variability in the MALDI-TOF MS signals, which was probably due to the manual deposition of the solution on the target [22]. Further improvement could be achieved with the automation of sample application.

**Fig. 5:**
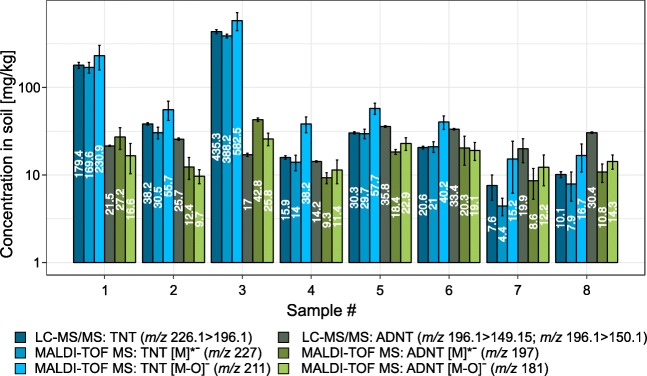
Matrix-assisted laser desorption/ionization time-of-flight mass spectrometry (MALDI-TOF MS) quantification results for the ion abundance of the radical anion [M]^•-^ and the fragment ion [M − O]^-^ of 2,4,6-trinitrotoluene (TNT; blue) and aminodinitrotoluenes (ADNT; green) of different soil extracts compared with the liquid chromatography–tandem mass spectrometry (LC–MS/MS) quantification results based on the multiple reaction monitoring of specific fragments. The LC–MS/MS ADNT concentration was calculated as the sum of 2-ADNT and 4-ADNT concentrations. Concentrations are given on a logarithmic scale. All measurements were done in triplicate

Extracts of contaminated soil were analyzed with the previously described MALDI-TOF MS method and a reference LC–MS/MS method. Details of the LC–MS/MS and MALDI-TOF MS results can be found in the electronic supplementary material. The soil samples investigated were contaminated with TNT, 2-ADNT, and 4-ADNT. Since the MALDI-TOF MS method is not able to distinguish isomers, the sum of the ADNT isomers is shown in Fig. 5 for both analytical approaches. MALDI-TOF MS results are depicted for the radical anion and the fragment ion. The results for TNT are quite similar for both methods. The quantification of the radical anion (*m*/*z* 227) resembled the results obtained with LC–MS/MS or gave slightly lower values. If the fragment ion (*m*/*z* 211) was used for quantification, higher TNT concentrations were determined. Further components of the soil matrix, such as organic matter or minerals, might have an effect on ionization and ISD of the analyzed explosive, resulting, for example, in increased fragmentation. Consequently, variances of explosive quantification in soil can be decreased through averaging the calculated concentrations of the radical and fragment anions. Conversely, extracts with a high TNT concentration (i.e., samples 1 and 3 in Fig. 5) tended to have ADNT concentrations similar to or slightly higher than the concentrations obtained by LC–MS/MS. For that reason, another approach was tested to increase the accuracy of the quantification.

### Calibration and quantification using multiple linear regression

**Fig. 6:**
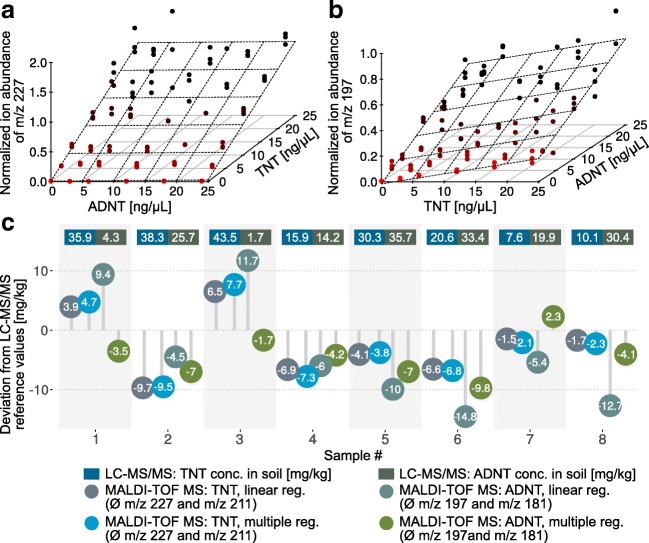
Multiple regression of matrix-assisted laser desorption/ionization time-of-flight mass spectrometry (MALDI-TOF MS) calibration data for **a** the 2,4,6-trinitrotoluene (TNT) peak at *m/z* 227 and **b** the aminodinitrotoluene (ADNT) peak at *m/z* 197. **c** Deviations from liquid chromatography–tandem mass spectrometry (LC–MS/MS) reference values for different soil extracts when simple and multiple linear regressions are used for calibration. LC–MS/MS reference values for each sample are given in milligrams per kilogram at the top. All measurements were done in triplicate

The LC–MS/MS multiple reaction monitoring optimization for TNT resulted reproducibly in a precursor ion with *m*/*z* 226.1 and a product ion with *m*/*z* 196.1, among other fragments (Table 1). The similarity of the product ion to the radical anion of ADNT (*m/z* 197) in MALDI-TOF MS led to the conclusion that there might be mutual interference of TNT with ADNT signals in standard mixtures and sample extracts. Consequently, we conducted multiple linear regression (classical least squares) with different TNT/ADNT ratios in the calibration mixture and plotted the normalized ion abundance of the peaks of interest in a 3D model (Fig. 6a,b). For the signal at *m*/*z* 227 a clear dependency on the TNT concentration can be observed, whereas the ADNT concentration had no effect on the ion abundance (Fig. 6a). These results were in accordance with the previous experiments. However, if the radical anion of ADNT (*m*/*z* 197) is examined, a dependency not only on ADNT concentration but also on TNT concentration is evidenced (Fig. 6b). The same behavior was observed for the fragment ions of TNT and ADNT (Fig. S5). Nevertheless, we used the data to conduct simple and multiple linear regressions and calculated the TNT and ADNT concentrations in different samples using both approaches. The results were compared and deviations from LC–MS/MS data calculated (Fig. 6c). As expected, there is no difference between simple linear regression and multiple linear regression for the calculation of TNT concentrations by MALDI-TOF MS. Mostly the values obtained by MALDI-TOF MS are lower than those determined by LC–MS/MS. This is the case not only for TNT but also for ADNT. However, a clear increase of accuracy is achieved if multiple linear regression is used for ADNT. Only sample 2 exhibits a slightly higher deviation from the reference value in comparison with the simple linear regression.

Hence, multiple linear regression can improve the MALDI-TOF MS quantification. But one has to keep in mind that the results of ADNT analysis depend on the correct calculation of the TNT concentration. If a lower TNT concentration is detected, it directly affects the calculation of ADNT concentrations (see samples 2, 4, 5, and 6 in Fig. 6c). Besides, it is also associated with a higher workload for calibration and this becomes quite difficult if more than two compounds are investigated.

### Evaluation of the MALDI-TOF MS method

Finally, we compared the most important features of an analytical method for the MALDI-TOF MS and the LC–MS/MS quantification of explosives in soil (Table 2). Both methods show a highly significant analyte specificity and concentration dependency (Table S6).

**Table 2:** Comparison of matrix-assisted laser desorption/ionization time-of-flight mass spectrometry (MALDI-TOF MS) and liquid chromatography–tandem mass spectrometry (LC–MS/MS) methods for the quantification of nitroaromatic explosives in soil

Parameter	MALDI-TOF MS	LC–MS/MS (ESI TQ^a^)
TNT linear range (ng/μL)	1–25	0.1–25
TNT lower limit of detection (ng/μL)	0.5	0.01
ADNT linear range (ng/μL)	1–50	0.01–1
ADNT lower limit of detection (ng/μL)	0.25	0.01
Concentration dependency (2-way ANOVA^b^)	***	***
Analyte specificity (2-way ANOVA^b^)	***	***
Distinction of isomers	Not possible	Possible
Preparation time after extraction per sample (min)	5	10
Sample filtration	Not necessary	Necessary
Acquisition time per sample (min)	<0.5	~20
Sample throughput	Very high	Low
Automation possibility	Easy	Difficult
Cost of consumables^c^ per sample (€)	~0.10	~1.50

^*aESI*^ electrospray ionization, *TQ* triple quadrupole

^b^significance level: *** *p* < 0.001

^c^Solvents, solutions (e.g., matrix), filtration equipment, vials, pipette tips

The advantages of LC–MS/MS include high reproducibility, a lower limit of detection, and the possible distinction of isomers. On the other hand, the preparation time and acquisition time are much longer than for the MALDI-TOF MS method. Besides, it is not suited for high throughput because of the material costs and the need for sample filtration. In contrast, MALDI-TOF MS can be easily adapted for automation. The small raw data files and simple data extraction support its applicability for high-throughput purposes. Even very high levels of contamination, for example, several grams of TNT per kilogram of soil [43], can be detected by MALDI-TOF MS without filtration of the extract, resulting in very high ion abundances (Fig. S6), which is not possible with LC–MS/MS. Therefore, high levels of contamination can be directly visualized by MALDI-TOF MS. For precise analysis it is still necessary to dilute the soil extract. However, the limit of detection for TNT of about 0.5 ng/μL in extracts, which corresponds to 1 mg/kg soil, is sufficient for the proposed industrial soil screening level of 96 mg/kg soil or even 21 mg/kg soil for residential soil screening [47] and far below the concentrations that can often be found in contaminated areas [1, 48]. Nevertheless, one has to keep in mind that no further separation techniques, such as chromatography, are applied and an only mass-based identification might lead to misinterpretation. Therefore, we recommend performing individual spot checks with other analytical methods, especially if the source of contamination is unknown. Furthermore, we used an extraction method that is fast and easy to use. To achieve higher accuracy, preconcentration using a salting-out step might be useful [26]. However, this would increase the manual effort and extend the extraction time considerably.

A desirable prospect of the MALDI-TOF MS method is the analysis of contaminated groundwater samples. A successful analysis seems likely, since the labile explosives are fixed through mixture with the organic matrix. The crystallization on the MALDI target might be prolonged because of the higher water content. Besides, water samples are often less contaminated, and preconcentration (salting-out or solid-phase extraction) might be necessary. Another prospect is the development or use of a portable MALDI-TOF mass spectrometer to allow on-site detection of explosives [49]. MALDI-TOF MS quantification of environmental contaminants can be further used for other explosives, such as RDX and HMX, or even other substance classes, such as chlorinated hydrocarbons, polycyclic aromatic hydrocarbons, and mineral oil residues. Further areas of application may be found for untargeted analysis. The special ionization properties of MALDI MS and simultaneous visualization of all sample ions in one spectrum can help to identify new degradation products or metabolites, which may be supported through ISD or MS/MS technologies.

Since the number of potentially contaminated sites in Europe (2.5 million) is more than seven times higher than the number of those under investigation (340,000), there is a great need for quick, low-cost detection methods [3]. Hence, the implementation of MALDI-TOF MS based environmental analytics may open new perspectives for fast and comprehensive screening of those areas and may likewise be fundamental for effective purification or remediation measures.

## Electronic supplementary material

ESM 1 (PDF 723 kb)
