# Supplementary material for: Quantification of nitroaromatic explosives in contaminated soil using MALDI-TOF mass spectrometry
Source: Anal Bioanal Chem. 2019 Jul 5;411(23):5993–6003. doi: 10.1007/s00216-019-01976-y (PMC6706601; doi:10.1007/s00216-019-01976-y)
Supplement: Supplementary file 1 — (PDF 723 kb) [file 216_2019_1976_MOESM1_ESM.pdf]

Analytical and Bioanalytical Chemistry

Electronic Supplementary Material

Quantification of nitroaromatic explosives in contaminated soil using MALDI-TOF mass spectrometry

S. Liane Kober, Henner Hollert, Marcus Frohme

Contents

Additional data of LC-MS/MS experiments

Fig. S1 ..... S3

Table S1 ..... S3

Additional data of MALDI-TOF MS experiments

Fig. S2 ..... S4

Table S2 ..... S5

Fig. S3 ..... S5

Fig. S4 ..... S6

Table S3 ..... S6

Table S4 ..... S7

Table S5 ..... S7

Fig. S5 ..... S8

Fig. S6 ..... S8

Table S6 ..... S8

Further information

Calculation of LOD and LOQ S9

Characteristics of the soil samples (Table S7) S9

Additional data of LC-MS/MS experiments

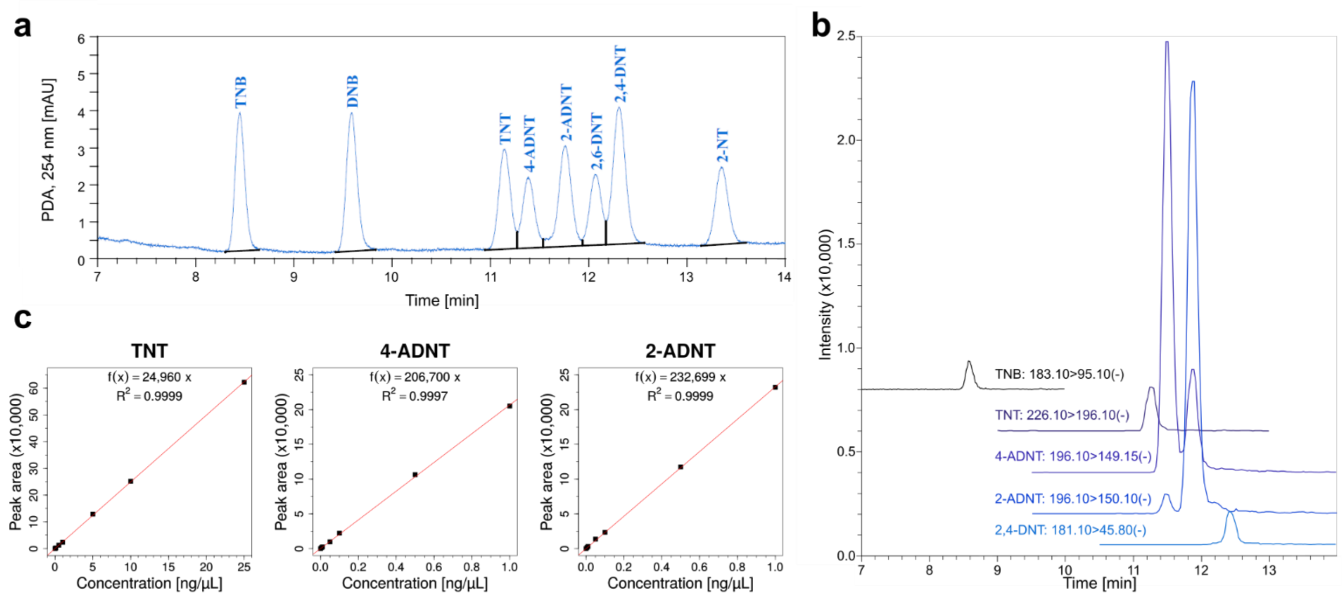

Fig. S1 PDA (a) and MRM (b) chromatograms of a LC-MS/MS standard mixture with a concentration of 1 ng/µL. TNB, TNT, 4-ADNT, 2-ADNT and 2,4-DNT show signals in both modes, whereas DNB, 2,6-DNT and 2-NT are only detectable with PDA. The MRM of 4-ADNT and 2-ADNT resulted in similar fragmentation patterns. Hence, the elution time is an additional important parameter for the correct differentiation of the isomers. c) Calibration curves for MRM quantification of TNT, 4-ADNT and 2-ADNT. The effective ionization of the ADNT isomers results in increased sensitivity but also a limited linear calibration range compared to TNT

Table S1 LC-MS/MS MRM quantification results for soil extracts that were contaminated with TNT, 4-ADNT and 2-ADNT. Diluted samples were measured in triplicates (a-c) and quantified using MRM mode. Detectable explosives were TNT with *m/z*: 226.10>196.10, 4-ADNT with *m/z*: 196.10>149.15 and 2-ADNT with *m/z*: 196.10>150.10

**Sample** [/]	**TNT conc.** [ng/µL]	**4-ADNT conc.** [ng/µL]	**2-ADNT conc.** [ng/µL]	**Dilution factor** [/]	**Final TNT conc.** [ng/µL]	**Final 4-ADNT conc.** [ng/µL]	**Final 2-ADNT conc.** [ng/µL]
1a 1:10	8.900	0.754	0.299	10	89.00	7.54	2.99
1b 1:10	8.316	0.787	0.297	10	83.16	7.87	2.97
1c 1:10	9.698	0.798	0.287	10	96.98	7.98	2.87
2a 1:10	1.916	0.925	0.330	10	19.16	9.25	3.30
2b 1:10	1.851	0.948	0.322	10	18.51	9.48	3.22
2c 1:10	1.971	0.992	0.336	10	19.71	9.92	3.36
3a 1:100	2.209	0.067	0.016	100	220.9	6.7	1.6
3b 1:100	2.050	0.071	0.012	100	205.0	7.1	1.2
3c 1:100	2.270	0.074	0.015	100	227.0	7.4	1.5
4a 1:10	0.813	0.584	0.112	10	8.13	5.84	1.12
4b 1:10	0.817	0.601	0.114	10	8.17	6.01	1.14
4c 1:10	0.749	0.604	0.119	10	7.49	6.04	1.19

5a 1:5	3.039	3.343	0.130	5	15.20	16.72	0.65
5b 1:5	2.930	3.486	0.132	5	14.65	17.43	0.66
5c 1:5	3.125	3.502	0.134	5	15.63	17.51	0.67
6a 1:5	2.126	3.256	0.116	5	10.63	16.28	0.58
6b 1:5	1.978	3.125	0.132	5	9.89	15.63	0.66
6c 1:5	2.063	3.242	0.142	5	10.32	16.21	0.71
7a 1:5	0.714	1.857	0.076	5	3.57	9.29	0.38
7b 1:5	0.771	1.888	0.092	5	3.86	9.44	0.46
7c 1:5	0.786	1.974	0.087	5	3.93	9.87	0.44
8a 1:5	0.922	2.879	0.107	5	4.61	14.40	0.54
8b 1:5	1.031	2.903	0.119	5	5.16	14.52	0.60
8c 1:5	1.079	2.993	0.124	5	5.40	14.97	0.62

Additional data of MALDI-TOF MS experiments

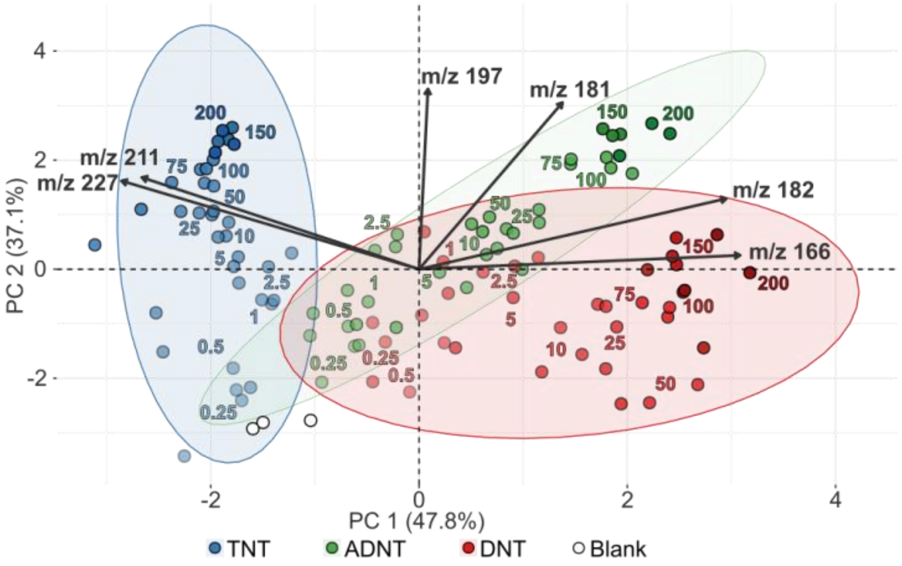

Fig. S2 Principal Component Analysis of MALDI-TOF MS spectra of TNT (blue), 2-/4-ADNT 1:10 (green) and 2,4-/2,6-DNT 1:1 (red) mixtures in different explosives concentrations concerning the ion abundance of distinct explosive signals. Concentrations are indicated as labels and given in ng/µL. To visualize the relative variation, we applied a log transformation to all normalized ion abundances before PCA. The PCA resulted in principal component 1 (PC 1) and PC 2 that account for 85 % of the data variance. Low concentrations of the individual explosives, which led to similar spectra, are located near the blank and in the left bottom corner of the PCA plot. On the contrary increasing concentrations resulted in distinct mass spectra. Thus, those individuals can be found in separate corners of the plot and indicate explosive specific signals

Table S2 Regression information of the calibration curves of distinct explosives peaks with calculated limits of detection (LOD) and limits of quantification (LOQ)

**Peak** [/]	**Equation** [/]	R^2 [/]	**LOD** [ng/µL]	**LOQ** [ng/µL]
m/z 137 $[NT]^{*-}$	f(x) = 0.0028x - 0.0057	0.9743	0.0003	0.0003
m/z 152 $[DNB\text{-}O]^{-}$	f(x) = 0.0197x + 0.0024	0.9991	0.0161	0.0371
m/z 166 $[DNT\text{-}O]^{-}$	f(x) = 0.028x + 0.0323	0.9978	0.0153	0.0293
m/z 168 $[DNB]^{*-}$	f(x) = 0.0215x + 0.0168	0.9984	0.0731	0.1571
m/z 181 $[ADNT\text{-}O]^{-}$	f(x) = 0.0392x + 0.0267	0.9992	0.0967	0.2367
m/z 182 $[DNT]^{*-}$	f(x) = 0.0504x + 0.0278	0.9986	0.0395	0.1025
m/z 197 $[ADNT]^{*-}$	f(x) = 0.0304x + 0.079	0.9969	0.1959	0.4969
m/z 211 $[TNT\text{-}O]^{-}$	f(x) = 0.0221x + 0.0551	0.9891	0.0549	0.1249
m/z 213 $[TNB]^{*-}$	f(x) = 0.0167x + 0.1259	0.9678	0.2441	0.5941
m/z 227 $[TNT]^{*-}$	f(x) = 0.0425x + 0.1340	0.9905	0.1965	0.4485

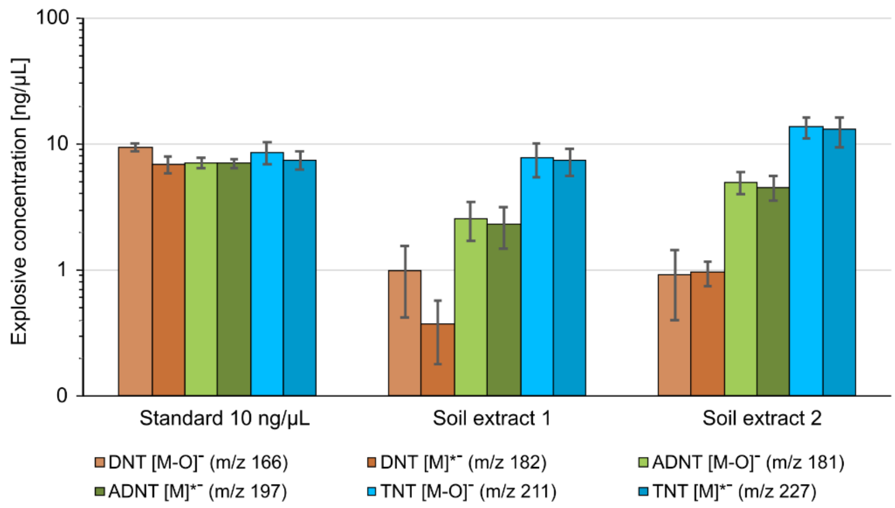

Fig. S3 MALDI-TOF MS quantification of a standard solution containing 10 ng/µL of TNT, ADNT 1:10 and DNT 1:1 and two soil extracts to determine the reproducibility of the method (n=8). The relative standard deviation (RSD) ranges from 6 to 19% for the analysis of the standard solution whereas the soil extracts exhibit an RSD of approximately 20% for the quantification of ADNT and TNT. The analysis of the low contamination with DNT in the soil extracts is also associated with a higher RSD of 50%.

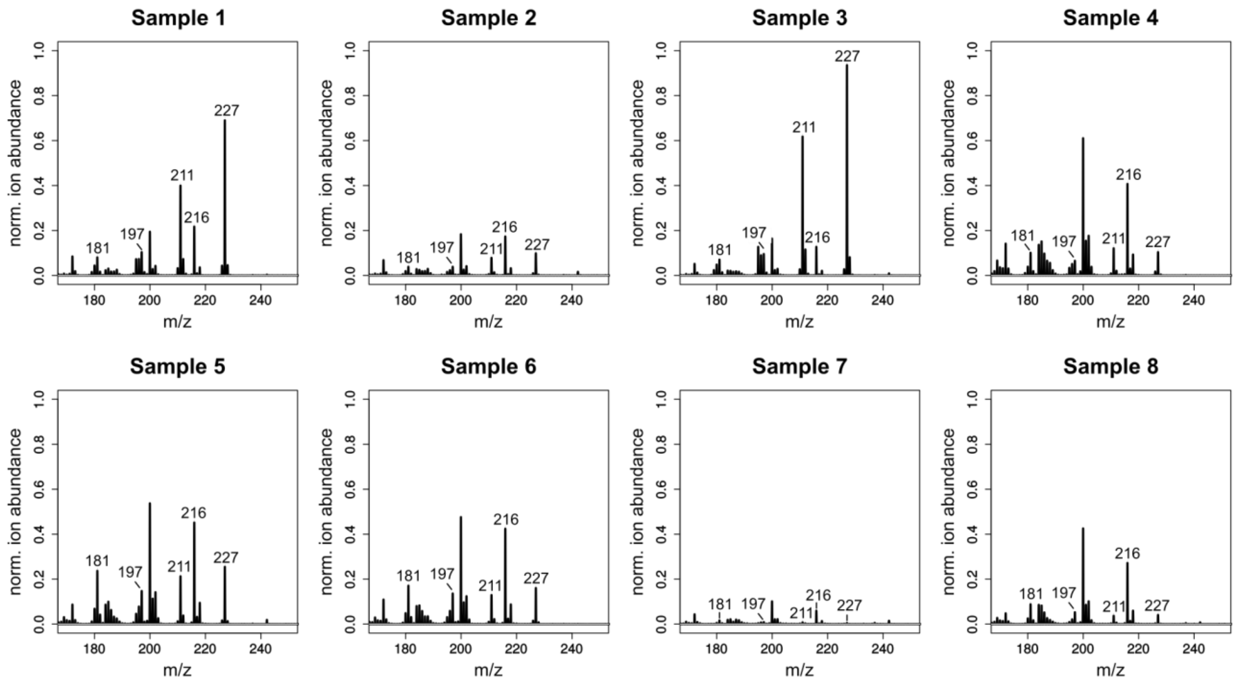

Fig. S4 Contaminated soil extracts that were analyzed with MALDI-TOF MS using the matrix DAN (20 mg/mL 80/20 ACN/H_2O) and the internal standard CDNT (25 ng/µL) in negative high-resolution reflectron mode (n=3). The spectra show different ion abundances for TNT (*m/z* 211, *m/z* 227) and ADNT (*m/z* 181, *m/z* 197). The normalization of those signals to the internal standard (m/z 216) and use of calibration curves leads to an effective quantification of explosives in soil

Table S3 MALDI-TOF MS quantification of soil extracts that were contaminated with TNT and ADNT. Samples were measured in triplicates (a-c) and concentrations calculated using the distinct explosives signals (*m/z* 227, *m/z* 211, *m/z* 197, *m/z* 181) after normalization to the internal standard (*m/z* 216) and calibration curves based on simple linear regression

**Sample** [/]	***m/z* 227 [TNT]$^{*-}$** [ng/µL]	***m/z* 211 [TNT-O]$^{-}$** [ng/µL]	***m/z* 197 [ADNT]$^{*-}$** [ng/µL]	***m/z* 181 [ADNT-O]$^{-}$** [ng/µL]
1a	91.61	100.49	14.24	7.42
1b	91.67	156.58	17.02	11.79
1c	71.11	89.28	9.53	5.71
2a	17.60	35.75	8.16	5.39
2b	15.19	22.94	4.79	3.83
2c	12.97	24.84	5.60	5.32
3a	183.43	219.76	22.33	15.31
3b	199.15	354.69	20.42	12.01
3c	199.68	299.30	21.43	11.38
4a	6.32	16.57	4.20	4.47
4b	6.05	17.10	4.31	4.96
4c	8.63	23.66	5.40	7.67
5a	14.35	30.18	9.23	12.65
5b	16.92	32.29	9.76	12.42
5c	13.26	24.13	8.54	9.22

6a	10.45	20.96	9.66	10.25
6b	9.04	16.26	6.73	7.03
6c	12.03	23.14	14.07	11.33
7a	2.52	3.61	3.65	5.17
7b	2.26	9.43	4.24	6.13
7c	1.83	9.79	4.98	7.03
8a	2.32	4.96	3.95	6.09
8b	4.46	10.25	6.42	8.63
8c	5.09	9.84	5.83	6.72

Table S4 Analysis of MALDI-TOF MS calibration data using simple and multiple linear regressions based on ADNT (a) and TNT concentrations (t) in a range from 0 to 25 ng/µL. The equations were used to calculate explosives concentration in soil sample extracts

Peak	Simple linear regression		Multiple linear regression	
	Equation	R^2	Equation	R^2
m/z 181 $[ADNT\text{-}O]^-$	f(a) = 0.045a – 0.0504	0.97	f(a,t) = 0.0268a + 0.116t – 0.0744	0.86
m/z 197 $[ADNT]^{*-}$	f(a) = 0.0269a – 0.0336	0.97	f(a,t) = 0.0154a + 0.112t – 0.0382	0.91
m/z 211 $[TNT\text{-}O]^-$	f(t) = 0.0473t – 0.0594	0.97	f(a,t) = 0.0007a + 0.0450t – 0.0613	0.93
m/z 227 $[TNT]^{*-}$	f(t) = 0.0557t – 0.0108	0.98	f(a,t) = -0.0001a + 0.545t + 0.1810	0.98

Table S5 Calculated explosives concentrations in soil samples using the equations for simple and multiple linear regression. Samples were measured in triplicates, concentrations calculated for the respective radical anion and fragment ion and finally averaged for determining the concentration of TNT and ADNT, respectively. Shown are the mean concentration and the standard deviation for each sample

**Sample**	**Simple linear regression**		**Multiple linear regression**	
[/]	**TNT concentration** [ng/µL]	**ADNT concentration** [ng/µL]	**TNT concentration** [ng/µL]	**ADNT concentration** [ng/µL]
1	19.91 ± 0.61	6.83 ± 0.88	20.31 ± 0.57	0.06 ± 0.65
2	14.27 ± 3.43	10.58 ± 2.10	14.37 ± 3.50	9.35 ± 1.44
3	25.00 ± 1.82	6.67 ± 0.53	25.59 ± 1.87	0.00 ± 0.48
4	4.46 ± 1.39	4.11 ± 0.86	4.27 ± 1.41	4.99 ± 0.97
5	13.11 ± 0.65	12.90 ± 1.76	13.27 ± 0.65	14.38 ± 2.42
6	6.99 ± 0.40	9.30 ± 1.69	5.88 ± 0.39	11.81 ± 2.37
7	3.05 ± 0.66	7.25 ± 0.80	2.73 ± 0.69	11.10 ± 2.02
8	4.18 ± 1.07	8.85 ± 1.78	3.90 ± 1.05	13.18 ± 2.46

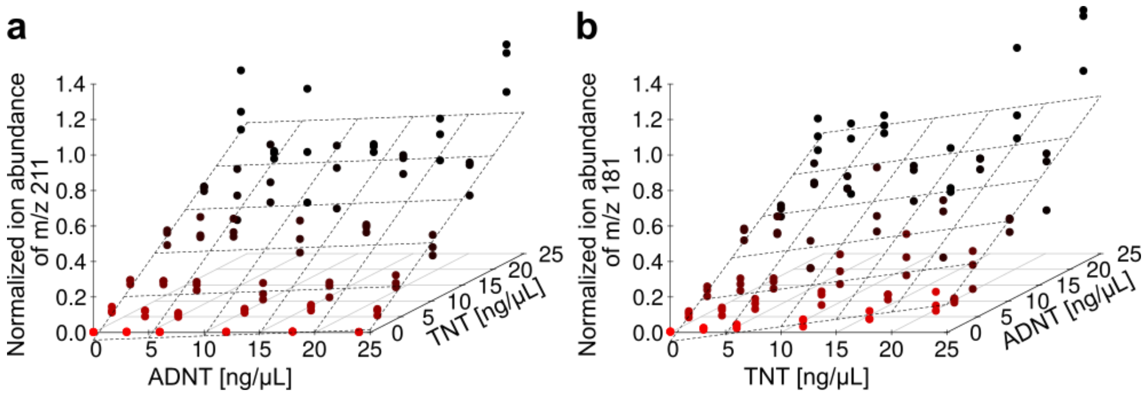

Fig. S5 Multiple regression of MALDI-TOF MS calibration data for a) TNT peak *m/z* 211 and b) ADNT peak *m/z* 181. Whereas *m/z* 211 shows almost no dependence on ADNT concentration, ADNT and TNT concentration have an impact on the ion abundance of *m/z* 181

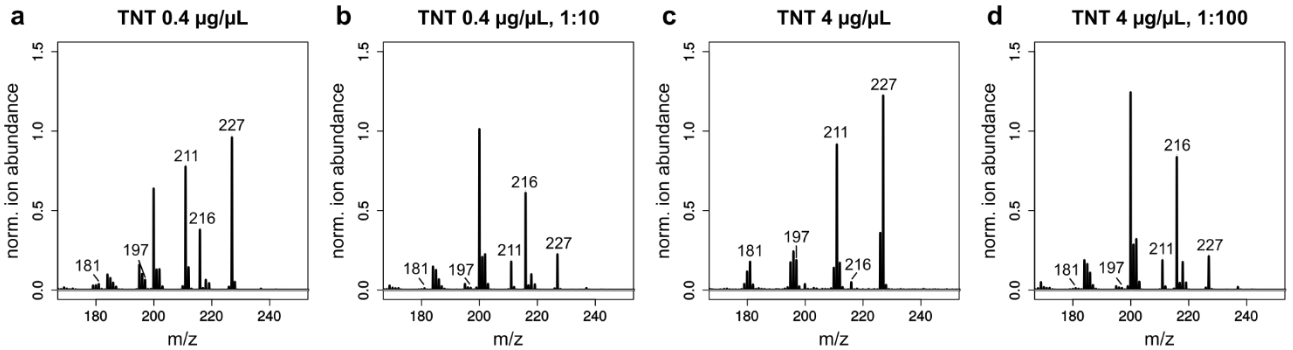

Fig. S6 Highly contaminated soil extracts that were analyzed with MALDI-TOF MS using the matrix DAN (20 mg/mL 80/20 ACN/H_2O) and the internal standard CDNT (100 ng/µL) in negative high-resolution reflectron mode (n=3). a) soil spiked with 800 mg TNT/kg resulting in an extract concentration of 400 ng/µL and b) a diluted sample (1:10), c) soil sample with a high contamination of 8 g TNT/kg soil (4,000 ng/µL) and d) a diluted sample (1:100). High TNT contaminations lead to very high signals of *m/z* 211 and *m/z* 227 compared to the internal standard (*m/z* 216) and can be detected without prior dilution, if no accurate results are required

Table S6 Two-way ANOVA analysis of LC-MS/MS and MALDI-TOF MS calibration data of TNT and ADNT. The analyte, concentration and the interaction of both factors have a highly significant effect on the signals of the respective method.

**LC-MS/MS**	**Sum of Squares**	**df**	**Mean Square**	**F**	**Significance (p value)**
Concentration	7.17E+13	1.00E+01	7.17E+12	5.51E+03	2.41E-92
Analyte	1.03E+13	2.00E+00	5.17E+12	3.97E+03	1.65E-69
Concentration * Analyte	2.90E+13	2.00E+01	1.45E+12	1.11E+03	1.50E-75
Error	8.59E+10	6.60E+01	1.30E+09		
Total	1.11E+14	9.80E+01			
**MALDI-TOF MS**					
Concentration	7.33E+01	9.00E+00	8.15E+00	1.22E+02	5.86E-43
Analyte	3.76E+00	3.00E+00	1.25E+00	1.88E+01	2.58E-09
Concentration * Analyte	8.16E+00	2.70E+01	3.02E-01	4.53E+00	7.23E-08
Error	5.33E+00	8.00E+01	6.67E-02		
Total	9.06E+01	1.19E+02			

Calculation of LOD and LOQ

The limits of detection were calculated according to the IUPAC definition using the mean ($\bar{x}_B$) and the standard deviation of the blank measures (sd_B):

$$LOD = \bar{x}_B + 3sd_B$$
$$LOQ = \bar{x}_B + 10sd_B$$

Characteristics of the soil samples

The analyzed soil samples were collected on a former TNT production site in Brandenburg. Table S6 summarizes the composition and depths of the soil samples.

Table S7 Composition and depths of the analyzes soil samples

**Sample** [/]	**Composition** [/]	**Depth** [cm]
1 and 2	Sand, no humus	0 – 14
3 and 4	Sand, little humus	16
5 and 6	Sand, rich in humus	26
7 and 8	Sand, little humus	40
